# Heterogeneity and potential therapeutic insights for triple-negative breast cancer based on metabolic‐associated molecular subtypes and genomic mutations

**DOI:** 10.3389/fphar.2023.1224828

**Published:** 2023-09-01

**Authors:** Lijuan Li, Nan Wu, Gaojian Zhuang, Lin Geng, Yu Zeng, Xuan Wang, Shuang Wang, Xianhui Ruan, Xiangqian Zheng, Juntian Liu, Ming Gao

**Affiliations:** ^1^ Department of Cancer Prevention Center, Tianjin Medical University Cancer Institute and Hospital, National Clinical Research Center for Cancer, Tianjin’s Clinical Research Center for Cancer, Key Laboratory of Breast Cancer Prevention and Therapy, Tianjin Medical University, Ministry of Education, Key Laboratory of Cancer Prevention and Therapy, Tianjin, China; ^2^ The Sixth Affiliated Hospital of Guangzhou Medical University, Qingyuan People’s Hospital, Qingyuan, China; ^3^ Department of Thyroid and Neck Tumor, Tianjin Medical University Cancer Institute and Hospital, National Clinical Research Center for Cancer, Tianjin’s Clinical Research Center for Cancer, Key Laboratory of Breast Cancer Prevention and Therapy, Tianjin Medical University, Ministry of Education, Key Laboratory of Cancer Prevention and Therapy, Tianjin, China; ^4^ Department of Phase I Clinical Trial, Tianjin Medical University Cancer Institute and Hospital, National Clinical Research Center for Cancer, Tianjin’s Clinical Research Center for Cancer, Key Laboratory of Breast Cancer Prevention and Therapy, Tianjin Medical University, Ministry of Education, Key Laboratory of Cancer Prevention and Therapy, Tianjin, China; ^5^ Department of Thyroid and Breast Surgery, Tianjin Union Medical Center, Tianjin Key Laboratory of General Surgery in construction, Tianjin Union Medical Center, Tianjin, China

**Keywords:** metabolic subtypes, triple-negative breast cancer, metabolic pathway, immune signature, immunotherapy response, mutation landscape

## Abstract

**Objective:** Due to a lack of effective therapy, triple-negative breast cancer (TNBC) is extremely poor prognosis. Metabolic reprogramming is an important hallmark in tumorigenesis, cancer diagnosis, prognosis, and treatment. Categorizing metabolic patterns in TNBC is critical to combat heterogeneity and targeted therapeutics.

**Methods:** 115 TNBC patients from TCGA were combined into a virtual cohort and verified by other verification sets, discovering differentially expressed genes (DEGs). To identify reliable metabolic features, we applied the same procedures to five independent datasets to verify the identified TNBC subtypes, which differed in terms of prognosis, metabolic characteristics, immune infiltration, clinical features, somatic mutation, and drug sensitivity.

**Results:** In general, TNBC could be classified into two metabolically distinct subtypes. C1 had high immune checkpoint genes expression and immune and stromal scores, demonstrating sensitivity to the treatment of PD-1 inhibitors. On the other hand, C2 displayed a high variation in metabolism pathways involved in carbohydrate, lipid, and amino acid metabolism. More importantly, C2 was a lack of immune signatures, with late pathological stage, low immune infiltration and poor prognosis. Interestingly, C2 had a high mutation frequency in PIK3CA, KMT2D, and KMT2C and displayed significant activation of the PI3K and angiogenesis pathways. As a final output, we created a 100-gene classifier to reliably differentiate the TNBC subtypes and AKR1B10 was a potential biomarker for C2 subtypes.

**Conclusion:** In conclusion, we identified two subtypes with distinct metabolic phenotypes, provided novel insights into TNBC heterogeneity, and provided a theoretical foundation for therapeutic strategies.

## 1 Introduction

In 2020, breast cancer become the main cause of malignant tumors and the fifth leading cause of death. Three million new patients and 685,000 deaths (Sung et al., 2021). As a disease with high heterogeneity, the treatment and prognosis of patients are greatly different. With the definition of breast cancer molecular subtypes being proposed, triple-negative breast cancer (TNBC) is classified as a type of breast cancer. This type has no expression of estrogen receptor (ER), progesterone receptor (PR) and human epidermal growth factor receptor 2 (HER2) (also known as ERBB2) (Goldhirsch et al., 2013; Waks and Winer, 2019). TNBC accounts for 10%–20%, with being prone to recurrence and metastasis. Due to the high early recurrence rate and limited treatment, the prognosis is very poor (Denkert et al., 2017; Garrido-Castro et al., 2019). Much effort has been devoted to classifying TNBC into subtypes of several molecular with different mutational characteristics and genomic changes (Bareche et al., 2018; Garrido-Castro et al., 2019; Jiang et al., 2021). Previous studies showed that cluster analysis identified TNBC subtypes, which provided new ideas for the treatment of TNBC (Lehmann et al., 2011; Jiang et al., 2019; Xiao et al., 2022).

Metabolic reprogramming, as an emerging hallmark, is a new tumor biomarker that plays a major role in the occurrence, progression, diagnosis, treatment, and prognosis (Martinez-Outschoorn et al., 2017; Xia et al., 2021). Due to the heterogeneous metabolic dependencies existing across different tumor types and even the same tissue (Hensley et al., 2016; Kim and DeBerardinis, 2019), we know little about the impact of tumor metabolic reprogramming on TNBC. In addition to some previous pan-cancer analysis (Rosario et al., 2018), the understanding of TNBC metabolic heterogeneity is still insufficient. Thanks to advancements in bioinformatics, we are now equipped to analyze high-throughput genetic data to gain insights into diseases, such as autoimmune disorders (Li et al., 2022; Li et al., 2023a; Li et al., 2023b) or cancers (Cheng et al., 2023; Tu et al., 2023). Building on this, our study delves into classifying Triple-Negative Breast Cancer (TNBC) from a metabolic perspective, shedding light on its underlying heterogeneity.

We used the screened metabolic genes to systematically check the diverse metabolic signatures of TNBC and identify two distinct metabolic subtypes. Differentially expressed genes (DEGs) were revealed by comparing transcriptome levels of patients with different subtypes. Subtyping TNBC Prognosis, metabolic characteristics, immune infiltration, clinical features, *in vivo* cell mutation characteristics, and drug sensitivity vary. Finally, a 100-gene classifier was designed and preliminarily verified to determine the classification of TNBC. This investigation may also provide insightful information into tumor-immune cell interactions, which retains tremendous potential for clinical therapeutic interventions in TNBC patients.

## 2 Materials and methods

### 2.1 Patients and samples

BRCA gene expression profiles were downloaded from five independent cohorts of patients, including TCGA-BRCA, GSE25066, GSE21653, GSE103091and METABRIC. Only samples from TNBC were reserved in all cohorts. Survival analysis only considered overall survival (OS) and disease-free survival (DFS). In the above five cohorts, METABRIC had no patient prognostic information. The remaining histological data were obtained from the TCGA-BRCA cohort, including copy number variant data obtained via firehose, and mutation MAF files obtained from the cBioPortal Pancancer Project. TCGA-BRCA partial samples of the predicted neoantigen numbers were obtained from published literature (Rooney et al., 2015). The metabolic gene file used for clustering (Possemato et al., 2011), the metabolism signatures (Rosario et al., 2018), the immune pathway signatures (Bindea et al., 2013) and the oncogenetic signature.txt (Sanchez-Vega et al., 2018) from different published literature. The drug information is from the GDSC database involving the drug’s R package “pRRophetic” for use in predicting the drug’s IC50. The external datasets were used to determine whether the defined subtypes are likely to respond to immunotherapy (Roh et al., 2017).

Limma package used for identifying DEGs (|log2FC| > 1 and *p* < 0.01). Genetic feature set files “c2.cp.kegg.v6.2.symbols.gmt” and “h,all,v60.2.symbols” were obtained from the Molecular Signature Database (MSigDB). Then, Clusterprofiler R package was used for Gene Set Enrichment Analysis (GSEA) (Yu et al., 2012).

To identify Aldo-Keto reductase family 1 member B10 (AKR1B10), we collected peripheral blood samples from 30 TNBC patients and 30 healthy individuals as controls from Tianjin Medical University Cancer Institute and Hospital (Tianjin, China) in 2022 for RT-qPCR, and their paraffin-embedded tissues for IHC. All patients were female patients who were recently admitted and had not undergone radiotherapy, chemotherapy, or surgery. Control group was determined to be free from TNBC and other malignant tumors.

### 2.2 Identification of TNBC subtypes through non-negative matrix factorization clustering

Because all data used in this study were derived from five platforms, and some of the data were normalized, we combined the data after normalizing each data using z-score to eliminate potential batch effects. We performed consensus NMF (Possemato et al., 2011) with 2-5 cluster numbers using TCGA data expression profiles and calculated the covariance coefficients for each decomposition. The MOVICS package (Lu et al., 2021) was used for differential expression analysis of these two subtypes, while the top 50 most significantly upregulated genes in each subtype were used as biomarkers for the different subtypes (*p* < 0.05, FDR < 0.25). In addition, we constructed a template using MOVICS.

### 2.3 Gene mutation analysis and single-sample gene-set enrichment analysis (ssGSEA)

Genomic variation analysis (GSVA) is a method genome augmentation, which calculates the characteristics of certain pathways or different populations based on expression spectra. The differences of gene sets between samples were investigated by GSVA R software package from relevant metabolic pathway gene sets (Rosario et al., 2018). Then, the limma package (Liu et al., 2019) was used to obtain the substitution gene scores, analyze the differences, and screen for DEGs features.

To identify the extent to which genes are up or downregulated within a single sample, ssGSEA is used for quantifying the immune composition of tumors. Here, we assessed the enrichment fraction of gene sets representing biological processes as well as biological pathways in bulk tumors or individual cancer cells by ssGSEA.

### 2.4 Detection of tumor microenvironment characteristics

The ESTIMATE algorithm (Yoshihara et al., 2013) can be applied to calculate the permeability and matrix content of immune cells (immune fraction) and stromal content (stromal fraction) of different subtypes, thus reflecting the microenvironmental characteristics of tumors. Microenvironmental Cell Population counter (MCPcounter) (Becht et al., 2016) was used for evaluating the penetration frequency of immune and non-immune cell populations in two subtypes.

### 2.5 Evaluation of genomic changes, number of new antigens, tumor mutation burden (TMB) and copy number variant (CNV) in different groups

The detection of co-occurrence and mutually exclusive mutations mainly relied on the CoMEt algorithm. Next, we predicted the different genotypes between different subtypes, including the number of neoantigens, TMB, copy number amplification, and the frequency of copy number deletions. We also performed an online analysis using GISTIC2 (Cibulskis et al., 2013) to obtain the number of amplifications and deletions for all samples and to calculate arm- and focal level somatic copy-number alterations (SCNAs) and G-scores in tumors with the input of “SNP6” files.

### 2.6 Prediction of treatment for each subgroup of immune checkpoint

MD-Anderson melanoma cohort treated with anti-CTLA-4 or anti-PD-1 is considered to be used to predict immunotherapy response (Roh et al., 2017). And then, we analyzed the sub map from the Genomics of Drug Sensitivity in Cancer (GDSC) database (Roh et al., 2017) and studied the sensitivity differences between the C1 and C2 groups after multiple drug treatments.

### 2.7 IHC staining

IHC staining was used to slice the dewaxed tissue portion of TNBC samples and cure with 3% hydrogen peroxide for a period of time. Block endogenous peroxidase for 30 min, then solidify with appropriate horseradish AKR1B10 antibody. The IHC fraction is calculated by multiplying the dyeing intensity by the percentage of cells. Definition of intensity: 0 (unstained), 1 (soft), 2 (medium), 3 (strong). Definition of percentage of cells: 1 (25%), 2 (26%–50%), 3 (51%–75%), and 4 (>75%). More than 3 was defined as positive, while less than or equal to 3 as negative.

### 2.8 RNA isolation and RT-qPCR

Triazole solution (AC0101-B; SparkJade, China) was used for extracting RNA from blood and tissues samples. 2×HQ SYBR qPCR Mix (ZF501; ZOMANBIO; Beijing, China) was used for PCR reaction. Primer sequences were listed in Supplementary Table S1. The levels of AKR1B10 expression were calculated by the method of 2−∆∆Cq.

### 2.9 Statistical analysis

R software (version 4.0.2) was used to process all data. Contingency table (χ2) variables used the chi‐square test and Fisher’s precision probability test for statistical significance. Kaplan-Meier method was used for survival analysis and compared the results by the log-rank test. Z test was used to assess whether there was a significant difference between the two groups. Univariate Cox proportional hazards regression models were used to assess the risk ratio for univariate analysis. A two-tailed *p*-value < 0.05 was considered statistically significant.

## 3 Results

### 3.1 NMF distinguishes two subtypes of TNBC

**FIGURE 1:**
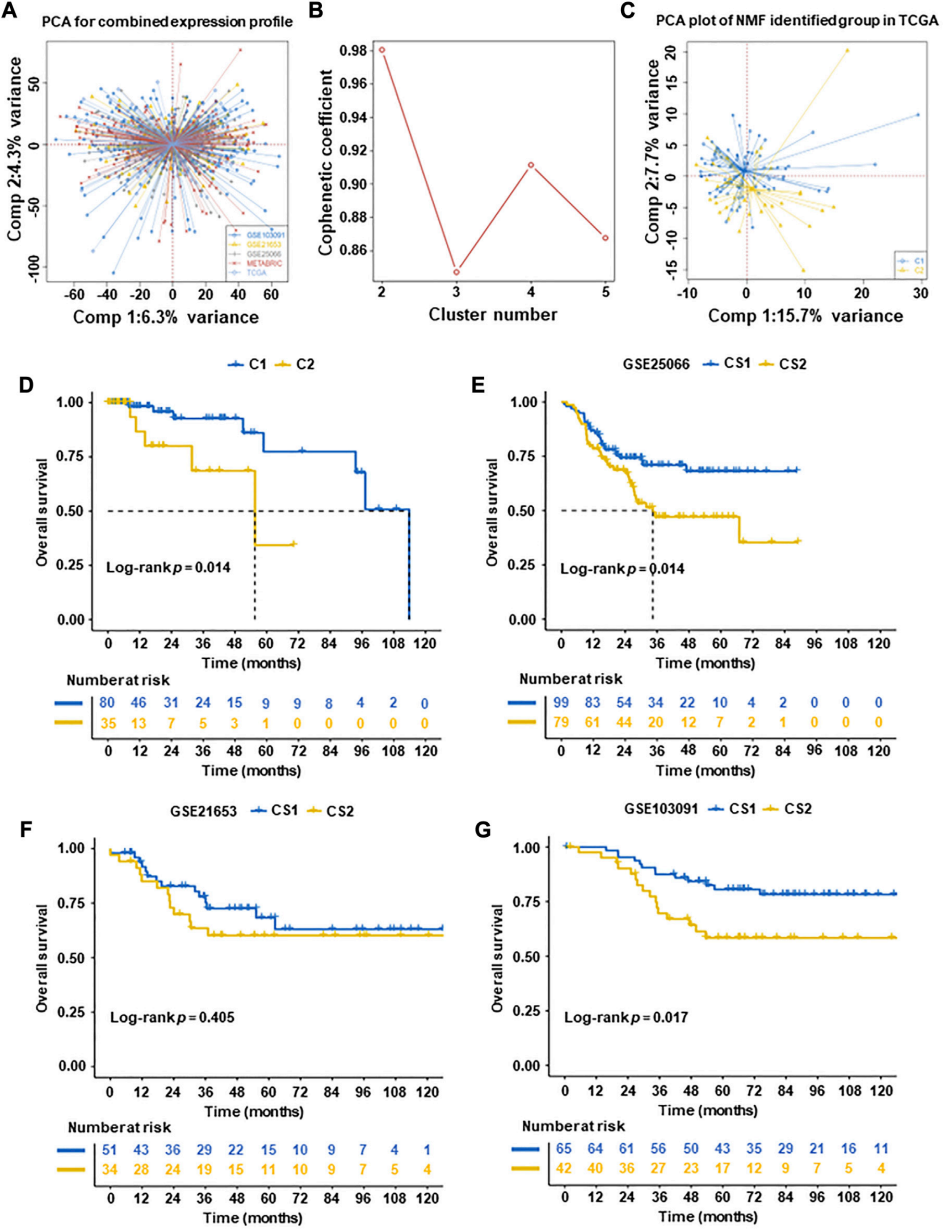
Subtyping of TNBC tumors according to non-negative matrix factorization (NMF) in five datasets. **(A)** Principal component analysis (PCA) of integrated expression profiles based on five TNBC datasets. **(B)** After comprehensive consideration, the optimal clustering number (k value) was 2. **(C)** PCA dimension reduction analysis was used to support the classification into two TNBC-subtypes. **(D)** Overall survival (OS) analysis of two subtypes in TCGA datasets. **(E–G)** Overall survival (OS) analysis of validation datasets (GSE25066, GSE21653, and GSE103091, excluding METABRIC with a lack of patient OS). The results of OS revealed that C1 had significantly better than C2 in TCGA datasets and validation datasets (GSE25066, GSE21653, and GSE103091) (*p* = 0.014, *p* = 0.014, and *p* = 0.017, respectively).

Based on TCGA database and NMF algorithm analysis, we divided TNBC into two subtypes with different metabolic characteristics. In this study, 115 cases of TCGA-TNBC patients were screened. Supplementary Table S2 showed clinical characteristics of TNBC patients. Before analyzing the TNBC NMF algorithm, we used the ComBat algorithm to eliminate batch processing effects in the TNBC queue. And after deleting the batch processing effect, draw a key element analysis diagram (Figure 1A). Previously, a total of 2,752 reported metabolic related genes (Possemato et al., 2011) were screened and downloaded as the basis for analyzing metabolomics in our study (Supplementary Table S3).

To identify subtypes in TNBC, Cox regression was used. A total of 637 prognostic genes were obtained (Supplementary Table S4). After a further adjusted *p*-value (*p* < 0.05), 277 candidate genes were identified (Supplementary Table S5). We then used the NMF algorithm to cluster the 277 candidate genes and drew the NMF with two to five sets (Figure 1B). After comprehensive consideration, the optimal clustering number (k value) was 2, defining two subtypes C1 (*n* = 80) and C2 (*n* = 35). To verify the consistency between subtype designations and two-dimensional distribution patterns, we reduced the PCA dimension (Figure 1C). Subsequently, the same conclusion was validated in the validation set (GSE25066, GSE21653, and GSE103091, excluding METABRIC with a lack of patient OS).

Finally, two TNBC molecular subtypes were established. We also used the survival information in the four queues to analyze the subtype survival of TNBC subsets. The OS of C1 was verified better than that of C2 in TCGA-TNBC patients (*p* = 0.014, Figure 1D) and other datasets patients (GSE25066 and GSE103091) (*p* = 0.014 and *p* = 0.017, respectively) (Figures 1E, G). No significant difference was observed in datasets (GSE21653) (*p* = 0.15) (Figure 1F).

### 3.2 Association of TNBC subtypes with metabolism-related signatures

In this study, we analyzed whether different TNBC subtypes have their own characteristics in distinct metabolic pathways. Firstly, we used the GSVA R package to score metabolic pathways (Rosario et al., 2018) (Supplementary Table S6). Limma difference test cross group was performed to confirm subtype-specific differential metabolic pathways, and heatmaps were constructed for visualization (Supplementary Figure S1).

**FIGURE 2:**
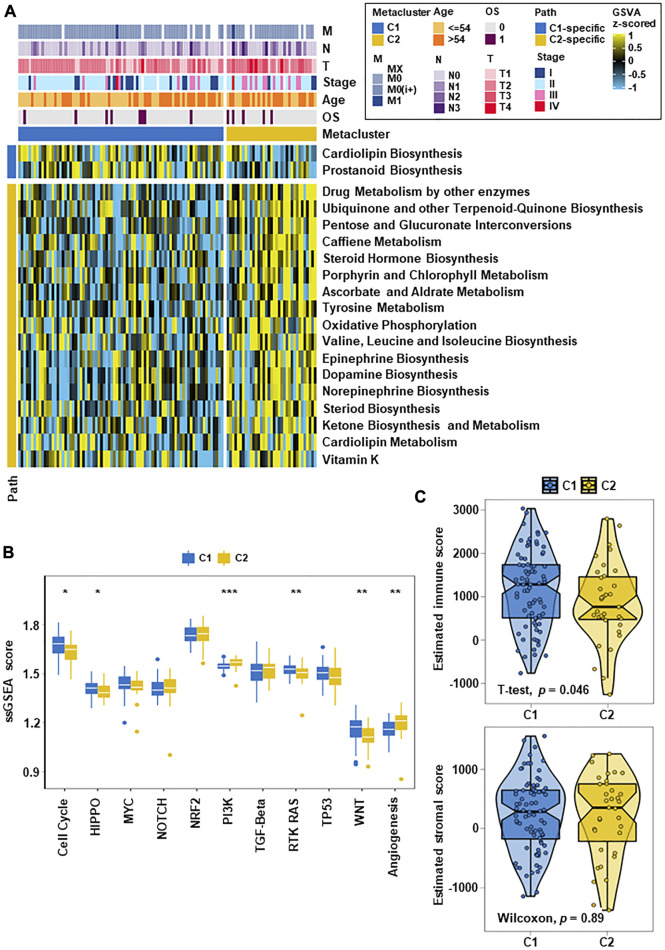
TNBC metabolic subtypes and tumor progression-related features. **(A)** Heatmap of metabolism-related features in the two subtypes. C1 and C2 had specific metabolic characteristics, with 2 metabolism-related pathways upregulated in C1 and 17 metabolism-related pathways significantly upregulated in C2. **(B)** Box plots of tumor progression-related signaling pathways in the two subtypes. After quantifying 11 carcinogenic pathways, the results showed differences between the them on multiple classic carcinogenic pathways. **(C)** Box line plots of the immune fraction and matrix fraction of ESTIMATE in the two subtypes (*p < 0.05, **p < 0.01, ***p < 0.001).

Furthermore, the DEGs between two groups were detected by GSVA enrichment again, and it was found that C1 and C2 had specific metabolic characteristics (Figure 2A). There were 17 metabolism-related pathways in C2 that were significantly upregulated, mainly involving pentose and glucuronate interconversion, steroid hormone biosynthesis, tyrosine metabolism, oxidative phosphorylation, ketone biosynthesis and metabolism. Similar outcomes that 17 metabolism-related pathways were activated in C2 were observed in the validation datasets (GSE25066, GSE21653, GSE103091 and METABRIC) (Supplementary Figures S2, S3). To determine the different activities of metabolic pathways, we represented the two subtypes in the TCGA-TNBC cohorts and validation cohorts and revealed that C2 contained the highest activation of metabolic pathways in all cohorts (Supplementary Figure S4).

In analyze the differences between the two subtypes in carcinogenesis-related pathways, we counted the GSVA enrichment points and plotted box-line plots. Eleven carcinogenesis-related pathways were selected and quantified. The results showed that different subgroups were closely related to the activation of different carcinogenic signaling pathways, which mainly involved cell cycle, PI3K, RTK-RAS, and angiogenesis (Figure 2B). C2 displayed significant activation of the PI3K and angiogenesis pathways. C1 had a stronger cell cycle, HIPPO, RTK-RAS and WNT signature than C2. These differences in carcinogenic pathway activity may affect their prognosis. After evaluating whether the subtype was related to the tumor microenvironment, it was found that the immune score of C1 was higher than that of C2 (*p* = 0.046), and the stromal score had no significant difference (Figure 2C).

### 3.3 Association of TNBC subtypes with immune infiltration

**FIGURE 3:**
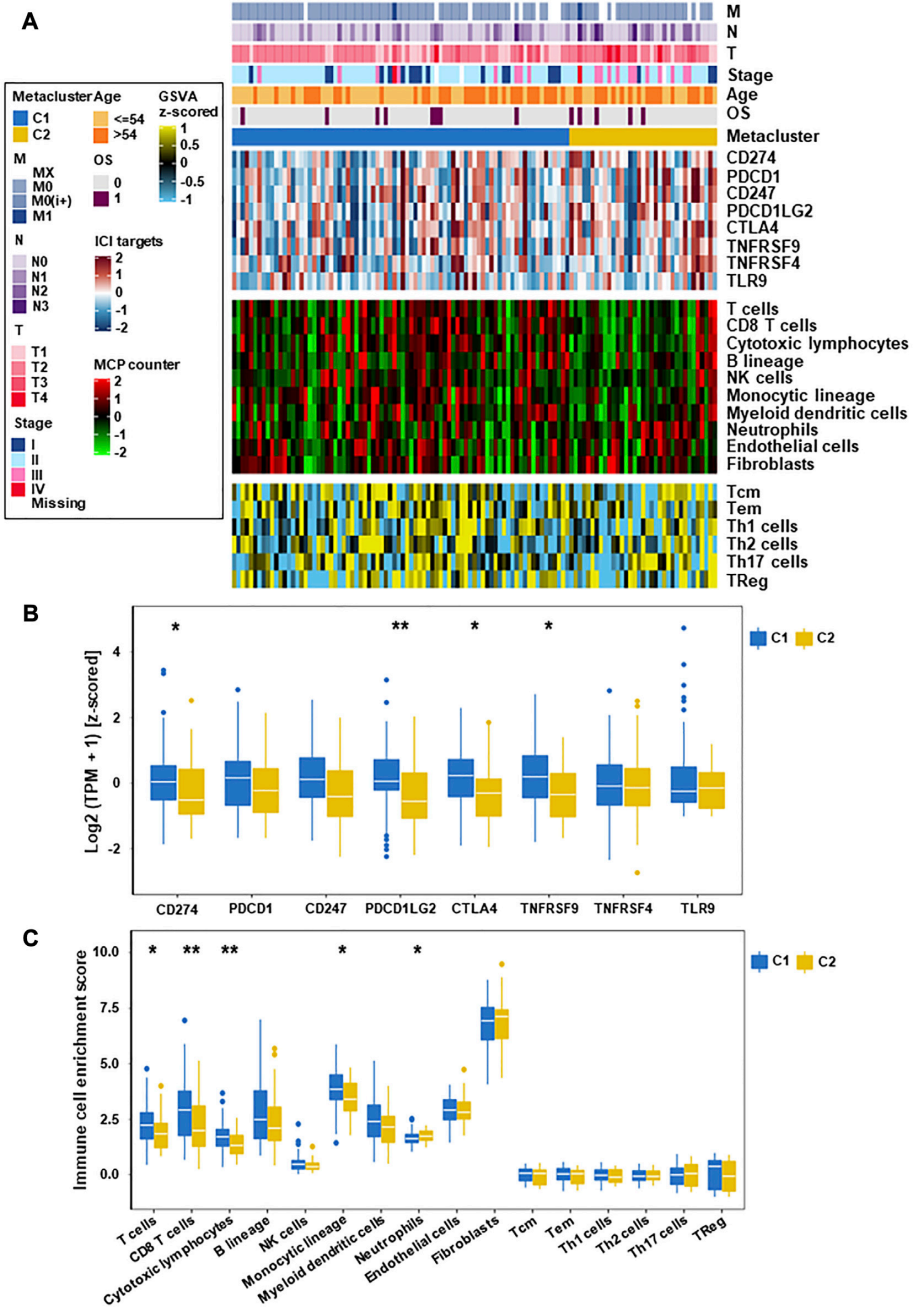
Immune characteristics of the two subtypes in the TCGA datasets. **(A)** Expression heatmap of immune cell and stromal cell populations in two TNBC subtypes. **(B)** Eight immune checkpoint genes in two TNBC subtypes. **(C)** Expression of different immune cells and stromal cells in C1 and C2 subtypes (*p < 0.05, **p < 0.01).

To evaluate the immune status of two subtypes, the MCP counter and ssGSEA algorithm were used to estimate the abundance of immune cells (Figure 3A). The results showed significant differences between different immune cell groups between the two subtypes (Figures 3B, C). Especially, the immune value of C2 in most immune cells was obvious lower than that of C1, except for neutrophils, fibroblasts and Th17 cells. According to this study, C1 was rich in more immune cells and had the highest immune score, which indicated that differences in the distribution of different immune cells may be the reason for the poorer prognosis of C2 than C1.

### 3.4 Association of TNBC subtypes with clinical features

To explore the relationship between these subtypes and clinical features, we analyzed the clinicopathological parameters between the two subtypes and constructed a clinical information heatmap of subtypes (Figure 3A). The results revealed that larger tumor size (*p* = 0.007) and advanced pathologic stage (TNM III/IV stage) (*p* = 0.001) were related to the C2 subtype (Supplementary Table S7). We also constructed a clinicopathological variables heatmap of subtypes in the validation cohorts and presented detailed data (Supplementary Figure S2; Supplementary Table S2). It is well known that larger tumor size and advanced TNM stage represent shorter survival in TNBC (Johansson et al., 2021).

### 3.5 Association of TNBC subtypes with mutations and created heatmaps for visualization

**FIGURE 4:**
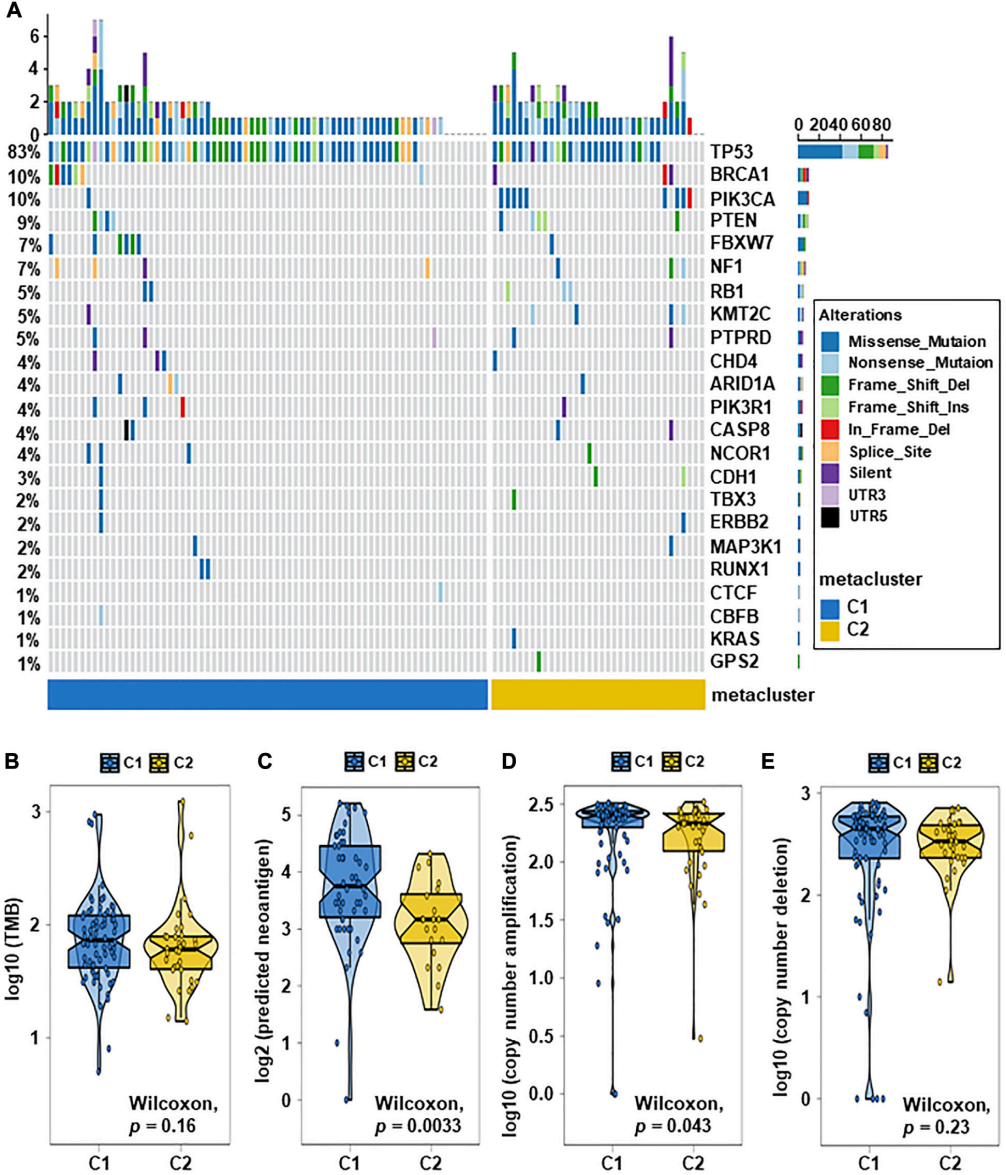
Relationship between TNBC subtypes and tumor mutation-related features. **(A)** Driver-type oncogenic mutations according to TCGA-TNBC typing with intragroup aggregation waterfall plots (see detailed statistical analysis in Supplementary Table S8). **(B)** Violin plots of gene mutations. There was a trend to show that the TMB of C1 was higher than that of C2, however there was no difference (*p* = 0.16). **(C)** Violin plots of predicted neoantigens. The quantity between two subtypes were significantly different (*p* = 0.0033). **(D,E)** Violin plots of copy number amplification and copy number deletion in TNBC subtypes. Patients within C1 only showed higher amplification than C2 (*p* = 0.043).

**FIGURE 5:**
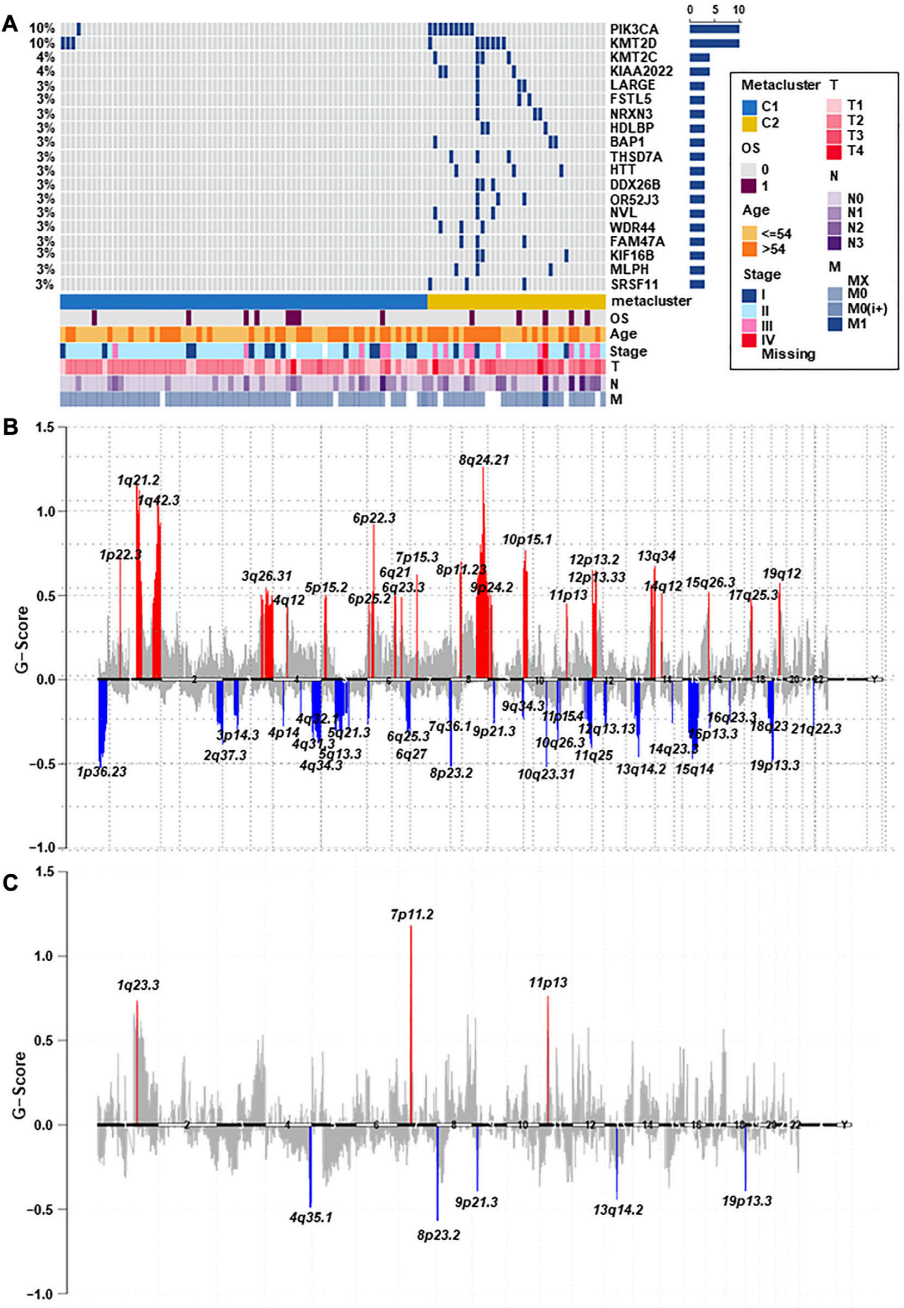
The landscape of somatic copy-number alterations in the two subtypes. **(A)** After adjusting *p* < 0.05, the genes with the most significant mutation frequencies between C1 and C2 groups were displayed. Specifically, C2 had a significantly higher mutation frequency of PIK3CA, KMT2D, KMT2C, and so on (see detailed statistical analysis in Supplementary Table S9). **(B,C)** Cytoband indicated differences in genomic copy-numbers between the two subtypes, with red representing amplification and blue representing deletion.

Breast cancer has been closely related to many genomic mutations in the body (Kim et al., 2021). To investigate the difference of somatic mutations frequency between TNBC subtypes, we applied specific driver mutations for breast cancer (Bailey et al., 2018) to estimate gene mutations and draw waterfall map. High mutation frequencies of TP53, BRCA1, PIK3CA, PTEN, FBXW7, NF1, RB1, KMT2C, and PTPRD in both TNBC subtypes were observed (Figure 4A; Supplementary Table S8). We found that C2 exhibited different mutation characteristics from C1. Specifically, C2 has a higher mutation frequency, such as PIK3CA, KMT2D, KMT2C, and so on (Figure 5A; Supplementary Table S9). We calculated the TMB for each metabolic subtype (Figure 4B). Although there was no difference (*p* = 0.16), a trend showed that the TMB of C1 was higher than that of C2. We also analyzed the total number of mutations and expected neoantigens (Figure 4C) and observed a significant difference between them (*p* = 0.0033). Subsequently, the frequency of amplification (Figure 4D) and deletion (Figure 4E) was showed and found that patients within C1 only showed higher amplification than C2 (*p* = 0.043).

Finally, we mapped a cell column to change the number of copies of each group by performing online GISTIC2.0 analysis, in which red represented gains and blue represented losses (Figures 5B, C). Both C1 and C2 observed copy number alterations in chromosome regions, including amplification at 11p13 and deletion at 8p23.2, 9p21.3, 13q14.2, and 19p13.3. In contrast to C1, C2 has significant amplification at 1q23.3 and 7p11.2. These differences could also explain that C2 has a better prognosis than C1. Therefore, changes in copy quantity might be the main mechanism behind the differences in metabolism and prognosis between the two group.

### 3.6 Specific sensitivity of TNBC subtypes for potential therapy

**FIGURE 6:**
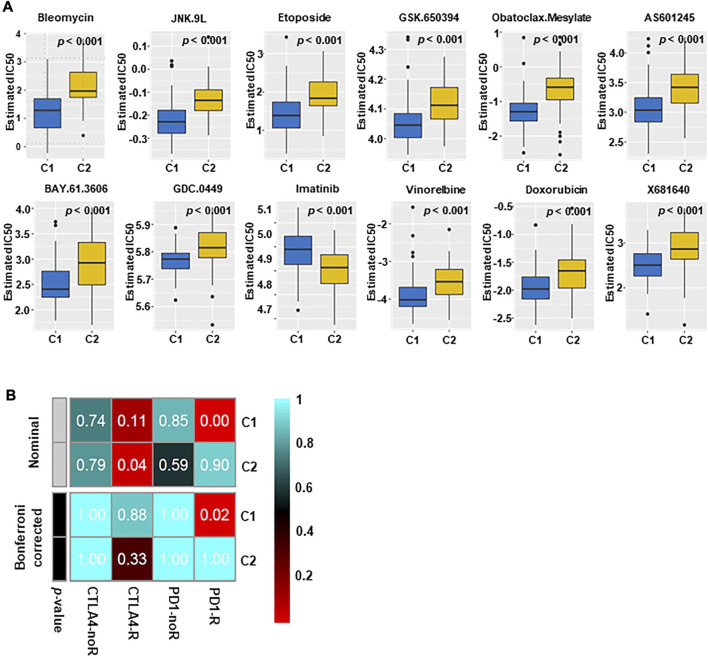
Immunotherapy and targeted therapy sensitivity of different subtypes. **(A)** The box plots of sensitivity to chemotherapy drugs in the two subtypes. The results indicated that C2 may not be sensitive to chemotherapy (All *p* < 0.001). **(B)** C1 may be more effective to PD1 inhibitors (Bonferroni correction, *p* = 0.02), and C2 may be more effective to CTLA4 inhibitors.

The difference in sensitivity to chemotherapeutic drugs and targeted drugs between two groups was analyzed by using the GDSC drug sensitivity database. The top 12 drugs with differential responses were plotted and listed (Figure 6A). After estimating the IC50 value, we found that C2 may be less sensitive to chemotherapy, including bleomycin, vinorelbine and doxorubicin (all *p* < 0.001).

The different immune infiltration patterns among TNBC subtypes suggested that further research on the response of immunotherapy was needed. To this aim, we matched the expression spectra of two subspecies to determine the similarity of the TCGA reaction spectra (Figure 6B). The results indicate that C1 may be more sensitive to PD1 treatment (*p* = 0.02), and C2 may have a better therapeutic effect on anti‐CTLA4.

### 3.7 Performance validation of one hundred‐gene classifier, and expression signature of Aldo-Keto reductase family 1 member B10 (AKR1B10)

**FIGURE 7:**
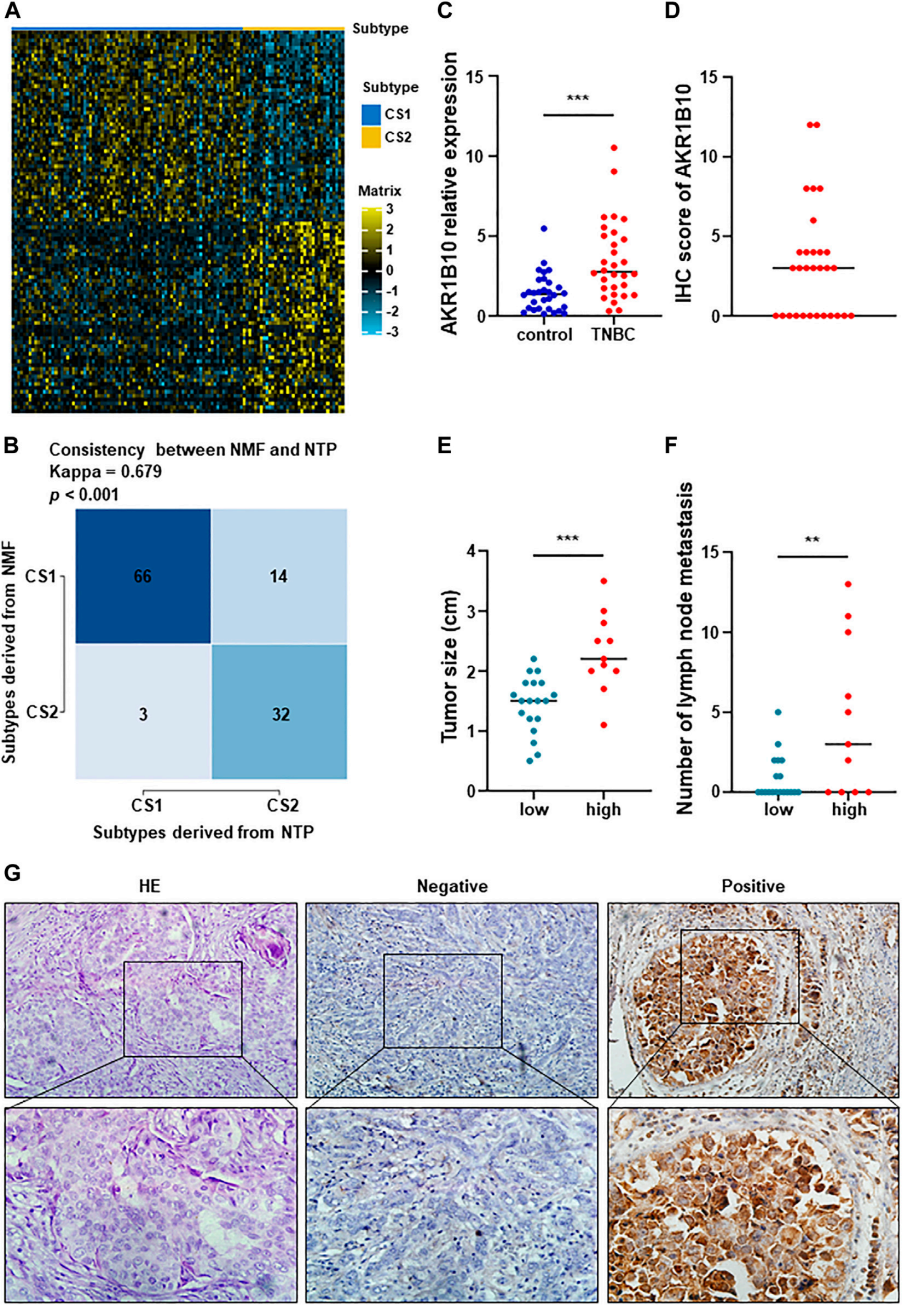
Performance validation of predictive metabolic-genes, and expression signature and preliminary validation of AKR1B10. **(A)** A 100‐gene classifier was composed of the top 50 genes with significant differences in each TNBC subtype, and visualized by a heatmap. **(B)** Constructing a 100 gene classifier for identifying TNBC classification. **(C)** Expression of AKR1B10 was significantly increased in peripheral blood of TNBC patients (***p < 0.0001). **(D)** IHC score of AKR1B10. **(E,F)** The difference of tumor size and number of lymph node metastasis between AKR1B10 positive group and AKR1B10 negative group (**p < 0.005). **(G)** AKR1B10 was significantly overexpressed in part of TNBC tissues. From left to right, they were HE staining, negative and positive respectively (×200 in the upper section, ×400 in the lower section).

And then, we extracted the top 50 genes of each metabolic specificity as biomarkers and constructed clinical models, and plotted correlation heatmaps using MOVICS (Bailey et al., 2018) package analysis. The classifier based on 100 genes was generated and visualized by heatmap (Figure 7A; Supplementary Table S10). In order to predict the identification of metabolic subtypes in each sample, we conducted consistency testing on the results of the two subtypes using the NTP algorithm and indicated that the characteristics of these genes can be replicated to determine the TNBC type (Figure 7B).

To better distinguish the two subtypes, we assume that AKR1B10 was an effective biomarker for C2. RT-qPCR and IHC staining were used to preliminarily verify this hypothesis. AKR1B10 were overexpressed in peripheral blood of TNBC patients than in healthy control (Figure 7C). IHC showed that AKR1B10 were positive in 11 cases of TNBC, with a positive rate of 36.7% (Figures 7D, G). The average tumor size of AKR1B10 positive group was 2.2 cm from 0.8 to 3.5 cm, which was higher than that of negative group (Figure 7E). In addition, the number of lymph node metastasis in AKR1B10 positive group were more than that in negative group (Figure 7F). Large tumor and many lymph node metastases often indicate poor prognosis of TNBC, which was consistent with C2 subtype. These results were listed in Supplementary Table S11.

## 4 Discussion

It is well known that the overall prognosis of TNBC is poor (Bianchini et al., 2016). With the increased understanding of metabolic reprogramming in breast cancer, traditional molecular characterization is no longer sufficient to fully elucidate tumor heterogeneity. As an important hallmark of tumors (Pavlova and Thompson, 2016; Pavlova et al., 2022), metabolic reprogramming may be beneficial to targeted therapy of TNBC. Recently, many TNBC classifications methods have been proposed, but a consensus on molecular taxonomy has not been reached. Thus, deeply exploring the metabolic characteristics and heterogeneity of TNBC is the key to providing provide precise treatment.

In this study, TNBC could be divided into two different metabolic related subtypes. Each subtype had different metabolic characteristics, prognoses, clinical features, tumor microenvironment characteristics, and so an. For C1, it was rich in immune signals and hardly involved in metabolic signals, gene expression was relatively high at immune monitoring points and scoring points. The increase of immunity and matrix indicated that these patients were allergic to drug allergy containing PD-1 inhibitor. In contrast, the C2 subtype displayed high variation in metabolism pathways involved in carbohydrate, lipid, and amino acid metabolism and a lack of immune signatures, with late pathological stage, weakened immunity and poor prognosis.

Our study indicated that C1 had abundant immune signatures and that C2 had overactivated metabolic related pathways. Considering the above results, we named C1 as the immune-related subtype and C2 as the metabolically active subtype. Subsequently, Patients in C2 had larger tumor size and later pathological stages, which implied that their overall prognosis were poor. The difference in metabolic characteristics and immune infiltration might be the important reason for the different prognoses of them. In this study, 17 associated metabolic pathways were significantly upregulated in C2, including pentose and glucuronate interconversions, oxidative phosphorylation, amino acid metabolism, steroid hormone and so on.

Previous studies have shown that glucose, amino acids and free fatty acids are important energy sources for tumor growth (Pavlova and Thompson, 2016), and metabolic disorders have a crucial impact on cancer (Micalizzi et al., 2021). Oxidative phosphorylation can promote distant metastasis and even induce chemotherapy resistance in TNBC (Davis et al., 2020; Evans et al., 2021). Tyrosine phosphorylation is an important mechanism for regulating signal transduction pathways and is also a common feature in oncogenic activation in cancer (Ostman et al., 2006; Taddei et al., 2020). Hence, the relationship between TNBC molecular subtypes may reveal the determining factors for TNBC metabolic differentiation classification.

Recently, more and more studies have confirmed that the tumor microenvironment (TME) plays an important role in the formation of breast cancer (Reis et al., 2018). Neoantigens can regulate the interaction between breast tumor cells and immune cells. This effect is presented by antigen-presenting cells (APCs) (Harbeck et al., 2019; Lhuillier et al., 2021). Although immune checkpoint inhibitors have achieved great advances in TNBC treatment, it is necessary to clearly distinguish which patients can benefit the most from this treatment (Li et al., 2021). Therefore, we compared the response to immune checkpoints of two TNBC-subtypes to obtain the potential significance of immunotherapy. Our results showed that C1 was significantly superior to C2 in both immune cell infiltration and neoantigens, which indicated that C1 has a higher response to treatment targeting immune checkpoints. Due to the inconsistent in carcinogenic signaling pathways, C1 may benefit from RAS inhibitors and WNT inhibitors in the future, while C2 may benefit from targeting PI3K and anti-angiogenesis. A series of studies have shown that targeting RAS, WNT, and PIK3 signaling pathways and angiogenesis are potential strategies to enhance the efficacy of cancer therapy (Verret et al., 2019; Xu et al., 2020).

In order to identify the molecular driving factors between two groups, we noticed that C2 had significant mutation frequencies in PIK3CA, KMT2D, KMT2C, and so on. Notably, C2 was accompanied by special chromosome copy number alterations, such as amplification at 7p11.2 and deletion at 9p21.3 and 13q14.2. Amplification at chromosome 7p11.2 (EGFR) can promote the invasion and metastasis of breast tumors (Chen et al., 2022). EGFR was overexpressed in metaplastic breast cancer, and EGFR inhibitor was potential therapeutic agent for metaplastic breast cancer with 7p11.2 amplification (Reis-Filho et al., 2006). Patients with 9p21.3 deletion and concomitant PIK3CA mutation were prone to recurrence and distant metastasis (Bartels et al., 2018). The mutation frequency of PIK3CA is only second to TP53 (Pascual and Turner, 2019). The same characteristics were obtained in our study.

Multiple studies have clarified that PI3K inhibitors are beneficial in enhancing the sensitivity of PIK3CA mutant TNBC to CDK4/6 inhibitors (Asghar et al., 2017), and have a good effect on HR+ breast cancer carrying PIK3CA mutations (Di Leo et al., 2018), which indicates the potential of combined targeted therapy. In this study, C2 was not sensitive to a variety of chemotherapies and immunotherapies, with high PI3K mutations and amplification at 7p11.2 (EGFR), suggesting that these TNBC patients may receive good treatment outcomes after receiving PI3K inhibitors or EGFR inhibitors. Previous studies have shown that When PIK3CA mutates, the glutamate pyruvate transaminase 2 in colorectal cancer (CRC) cells is significantly upregulated, thereby affecting the reprogramming of glutamine metabolism (Hao et al., 2016). The metabolites of glutamine can be used not only to produce ATP, but also to synthesize certain macromolecules to promote tumor formation. For example, the ATP concentration and ATP/ADP ratio in PIK3CA mutant cells were higher. Mutations in PIK3CA in adipose tissue can lead cells to acquire many characteristic changes of cancer cells, such as increased glucose uptake, enhanced Warburg effect activity, and increased synthesis of oncogenic macromolecules (Ladraa et al., 2022). KMT2D mutations can significantly alter the biosynthesis of various metabolic products within cells, such as aerobic glycolysis and β- Oxidation, degradation, and uptake of lipids (Koutsioumpa et al., 2019). The above results indicate that these gene mutations can promote the differentiation of C2 subtypes by affecting metabolic reprogramming. Meanwhile, once these genes undergo mutations, they will further promote tumor progression by altering the activity of glucose and lipid metabolism in C2 patients. This may be the root cause of poor prognosis in C2 patients.

Our study had some limitations. First, bioinformatic analysis of metabolic and genomic alterations failed to pinpoint the precise cause of the difference in prognosis between the two subtypes. Second, the two subtypes classified according to immune and metabolic conditions need to be functionally validated further. Furthermore, the sensitivity of different subtypes of drugs must also be validated through clinical trials to explore the feasibility of translating these results into clinical practice. Finally, although it was preliminarily verified to identify the subtypes of TNBC, data from multiple centers and large samples will be needed to support this conclusion in the future.

## 5 Conclusion

In summary, this study revealed differences in TNBC metabolism and identified two subtypes. Subtype C1 was abundant in immune signatures but barely active in metabolic signatures, with higher gene expression at immune checkpoints and higher immune and matrix scores. This indicated that the C1 was allergic to PD-1 inhibitors. Subtype C2, on the other hand, had a high variation in metabolic pathways and a lack of immune signatures, as well as late pathological stage, low immune infiltration and poor prognosis. By dividing TNBC into two clusters, this study elucidated the reasons for the differences in prognosis of TNBC from the perspectives of metabolism and immune response. For the first time, we proved that C1 may be more sensitive to immunosuppressive drugs. RAS inhibitors and WNT inhibitors, whereas C2 may benefit from targeting PI3K and anti-angiogenesis. Furthermore, AKR1B10 based on the one hundred-gene classifier was a potential biomarker for identifying C2 subtypes. This provides a theoretical basis for further rationalizing TNBC subtypes to provide precise therapeutic strategies.

## Data Availability

The datasets presented in this study can be found in online repositories. The names of the repository/repositories and accession number(s) can be found in the article/Supplementary Material.

## Supplementary material

SUPPLEMENTARY FIGURE S1 Heatmap of metabolic related characteristics in TCGA cohorts. Univariate Cox regression was used to identify metabolic related genes related to OS and visualized with heatmap.

SUPPLEMENTARY FIGURE S2 TNBC metabolic subtypes and tumor progression-related features in validation datasets. Heatmap of all metabolism-related genes were structured between two subtypes in the GSE25066 cohorts **(A)**, GSE21653 cohorts **(B)**, GSE103091 cohorts **(C)** and METABRIC cohorts **(D)** to verify the initial classification in TCGA cohorts. The results showed that the classification was consistent.

SUPPLEMENTARY FIGURE S3 Association between 17 metabolism-related signatures and the TNBC subtypes in validation datasets. There were 17 metabolism-related pathways in C2 that were significantly upregulated in GSE25066 cohorts **(A)**, GSE21653 cohorts **(B)**, GSE103091 cohorts **(C)** and METABRIC cohorts **(D)**.

SUPPLEMENTARY FIGURE S4 Validation of the differential activity of metabolic pathways between the two subtypes. The nearest template prediction (NTP) indicated two subtypes in TCGA-TNBC cohorts and validation cohorts, and demonstrated that C2 subtypes from five cohorts had stronger metabolic activity. Representing the two subtypes in the TCGA-TNBC cohorts **(A)**, GSE25066 cohorts **(B)**, GSE21653 cohorts **(C)**, GSE103091 cohorts **(D)** and METABRIC cohorts **(E)**.

## Abbreviations

TNBC, Triple-negative breast cancer; TCGA, The Cancer Genome Atlas; NMF, Non-negative matrix factorization; DEGs, Differentially expressed genes; PIK3CA, Phosphatidylinositol-4,5-bisphosphate 3-kinase catalytic subunit alpha; KMT2D, Lysine methyltransferase 2D; KMT2C, Lysine methyltransferase 2C; GEO, Gene Expression Omnibus; GDC, Genome Data Commons; OS, overall survival; DFS, Disease-free survival; MSigDB, Molecular Signature Database; GSEA, Gene Set Enrichment Analysis; GSVA, Genomic variation analysis; ssGSEA, Single-sample gene-set enrichment analysis; TMB, Tumor mutation burden; CNV, Copy number variant; GDSC, Genomics of Drug Sensitivity in Cancer; PD-1, Programmed cell death-1; PD-L1, Programmed cell death ligand-1; CTLA-4, Cytotoxic T-lymphocyte antigen-4; AKR1B10, Aldo-Keto reductase family 1 member B10.
