# Supplementary figures and images for: Heterogeneity and potential therapeutic insights for triple-negative breast cancer based on metabolic‐associated molecular subtypes and genomic mutations
Source: Front Pharmacol. 2023 Sep 1;14:1224828. doi: 10.3389/fphar.2023.1224828 (PMC10502304; doi:10.3389/fphar.2023.1224828)

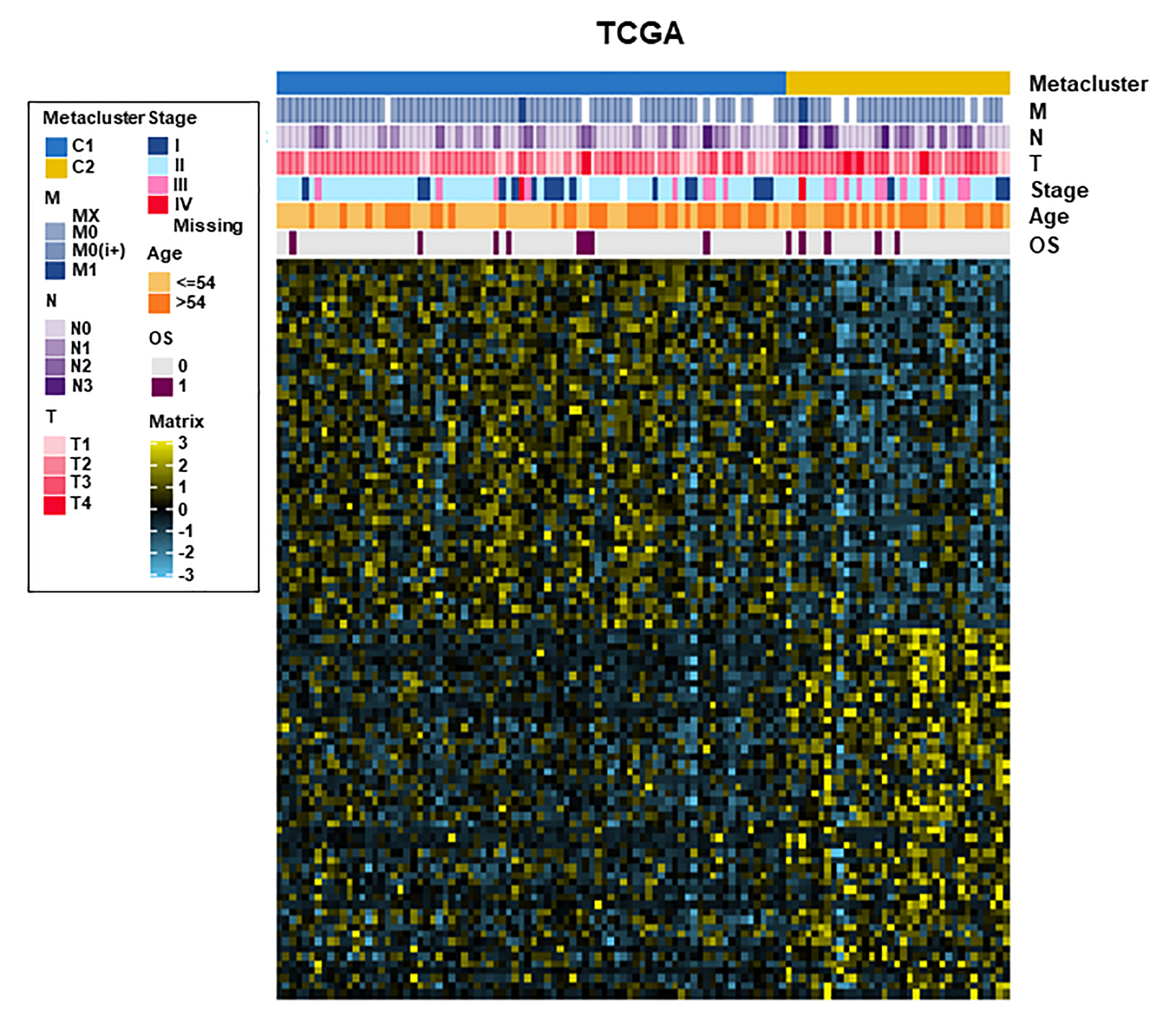

Supplement: Supplementary file 1 [file Presentation1.zip › Figure S1.tif]

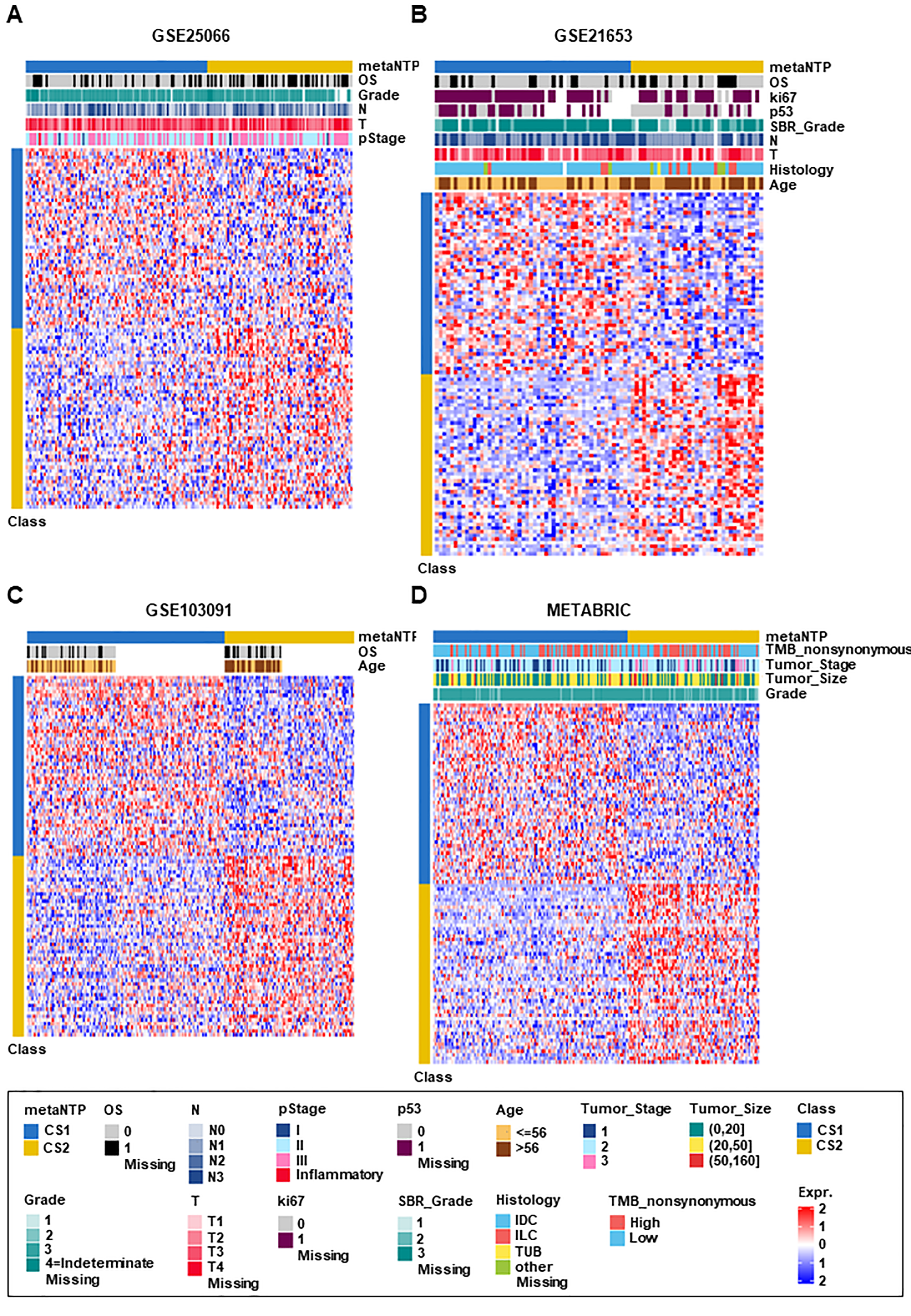

Supplement: Supplementary file 1 [file Presentation1.zip › Figure S2.tif]

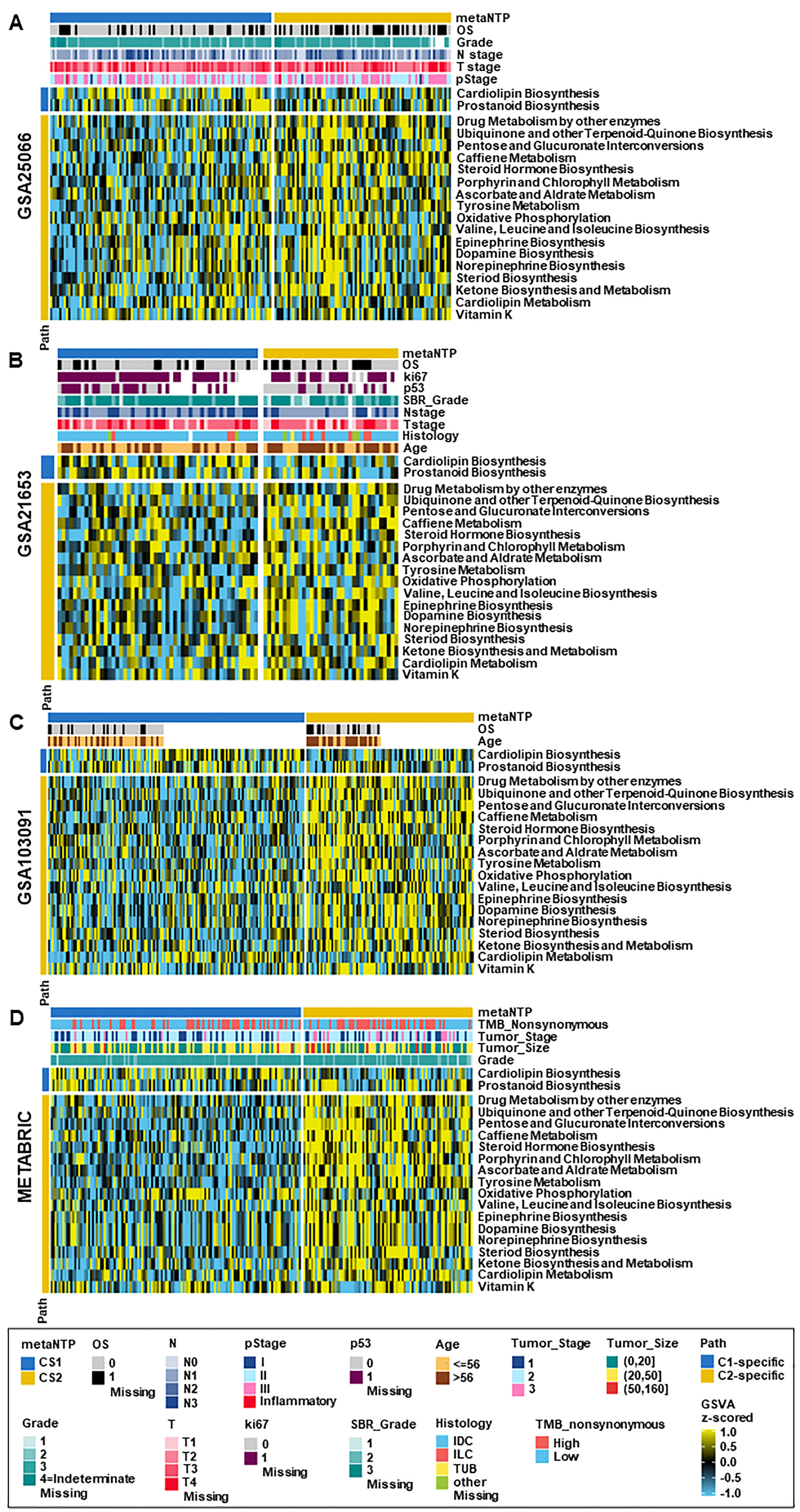

Supplement: Supplementary file 1 [file Presentation1.zip › Figure S3.tif]

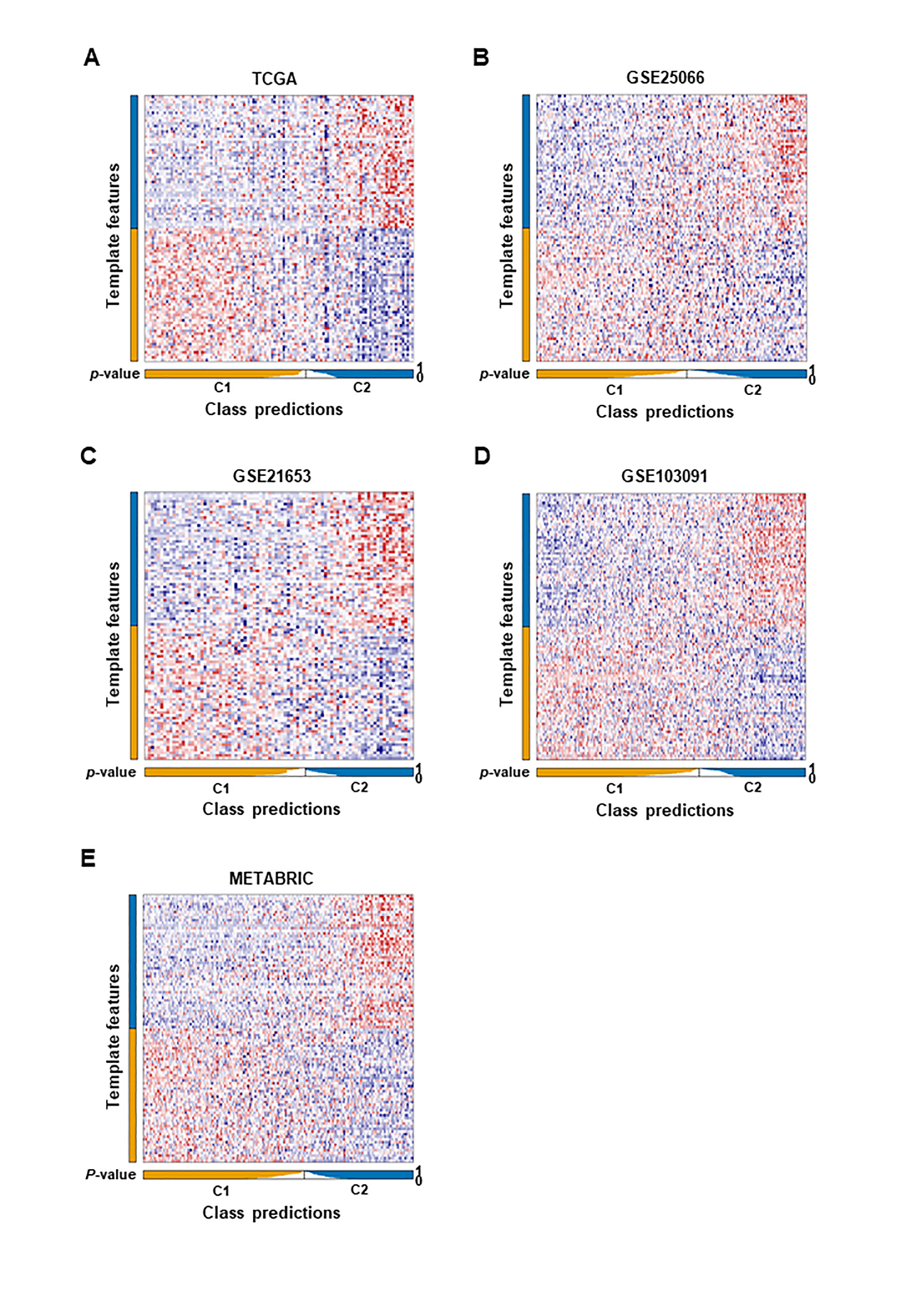

Supplement: Supplementary file 1 [file Presentation1.zip › Figure S4.tif]
